# Degradation of Root Community Traits as Indicator for Transformation of Tropical Lowland Rain Forests into Oil Palm and Rubber Plantations

**DOI:** 10.1371/journal.pone.0138077

**Published:** 2015-09-14

**Authors:** Josephine Sahner, Sri Wilarso Budi, Henry Barus, Nur Edy, Marike Meyer, Marife D. Corre, Andrea Polle

**Affiliations:** 1 Department for Forest Botany and Tree Physiology, Büsgen-Institute, Georg-August University Göttingen, Göttingen, Germany; 2 Department of Sylviculture, Faculty of Forestry, Jalan Lingkar Akademik Campus, IPB Darmaga, Bogor, Indonesia; 3 Department of Agrotechnology, Faculty of Agriculture, Tadulako University, Palu, Indonesia; 4 Institute for Geography, Georg-August University Göttingen, Göttingen, Germany; 5 Department for Soil Science of Tropical and Subtropical Ecosystems, Büsgen-Institute, Georg-August University Göttingen, Göttingen, Germany; Institution and Department: Université de Sherbrooke, CANADA

## Abstract

Conversion of tropical forests into intensely managed plantations is a threat to ecosystem functions. On Sumatra, Indonesia, oil palm (*Elaeis guineensis*) plantations are rapidly expanding, displacing rain forests and extensively used rubber (*Hevea brasiliensis*) agro-forests. Here, we tested the influence of land use systems on root traits including chemical traits (carbon, nitrogen, mineral nutrients, potentially toxic elements [aluminium, iron] and performance traits (root mass, vitality, mycorrhizal colonization). Traits were measured as root community-weighed traits (RCWTs) in lowland rain forests, in rubber agro-forests mixed with rain forest trees, in rubber and oil palm plantations in two landscapes (Bukit Duabelas and Harapan, Sumatra). We hypothesized that RCWTs vary with land use system indicating increasing transformation intensity and loss of ecosystem functions. The main factors found to be related to increasing transformation intensity were declining root vitality and root sulfur, nitrogen, carbon, manganese concentrations and increasing root aluminium and iron concentrations as well as increasing spore densities of arbuscular mycorrhizas. Mycorrhizal abundance was high for arbuscular and low for ectomycorrhizas and unrelated to changes in RCWTs. The decline in RCWTs showed significant correlations with soil nitrogen, soil pH and litter carbon. Thus, our study uncovered a relationship between deteriorating root community traits and loss of ecosystem functionality and showed that increasing transformation intensity resulted in decreasing root nutrition and health. Based on these results we suggest that land management that improves root vitality may enhance the ecological functions of intense tropical production systems.

## Introduction

Globally, tropical rain forests are rapidly converted to plantation agriculture [1]. In Indonesia, which is together with Malaysia the world´s largest producer of palm oil [2], 40% of the forest (64 Mio ha) was lost since the countries´ independence in 1945 [3]. In the 1950s rubber (*Hevea brasiliensis*) was introduced as a crop tree and is currently cultivated in two systems, in intense monocultures often with high yielding clones (rubber plantation) or as jungle rubber. Jungle rubber is a complex, extensive form of agro-forestry, usually established after swidden agriculture, where rubber trees are grown together with naturally established secondary forest [4,5]. Tree species richness is lower but the forest structure of jungle rubber is similar to that of unmanaged lowland rain forests [4–6]. Pristine lowland rain forests exist only in fragments and most unmanaged forests, even in protected areas, are secondary forests. Since the 1990s with the introduction of oil palms (*Elaeis guineensis*), expansion of plantation area at the expense of primary and secondary forests has drastically increased [7], with particularly high rates (> 2% per year) on Sumatra [8]. Because of the world´s increasing demand for biofuel, chemical raw materials and edible oil, palm oil production is now a major driver for tropical forest conversion [2]. The ecological consequences of this rapid transformation process are severe, including for example massive loss in biodiversity, soil degradation, reduction in carbon storage, decreased energy flux, increases in greenhouse gas emissions, etc. [9–12]. While the alterations of above-ground ecosystem properties and processes have been intensively studied, much less is known about the below-ground plant responses to these massive changes.

Roots together with their associated mycorrhizal fungi play a central role for nutrient uptake and allocation to the above-ground parts; they further mediate carbon transfer to the soil, thereby, eventually affecting biogeochemical cycles [13–16]. In tropical forests, most tree species including the introduced rubber and oil palms form symbioses with arbuscular mycorrhizal (AM) fungi, but in lowland tropical forests also a number of native species occur, e.g. dipterocarps that associate with ectomycorrhizal (EM) fungi [17].

The ability of tree roots to form mutualistic AM or EM associations is a typical species-related trait that can mediate differences in plant nutrition, especially of phosphorus and nitrogen [18]. Root functional traits have often been studied in agroecological systems [19], but only little information is available for forest trees, especially regarding the chemical root traits. In tropical ecosystems with potentially 100s of species per hectare [5,6] *in situ* root traits are difficult to measure, because a trait is defined as a feature of a species [20]. Instead, information on root traits can be gathered at the community level of the co-occurring species and can then be defined as “root community-weighed traits” (RCWTs). Only few studies addressed the variation of RCWTs. Prieto et al. [21] found that root morphology, a trait related to resource acquisition and root litter degradability, a trait indicating conservation of resources, co-varied for root communities with land use across tropical, Mediterranean and montane climate. In grassland ecosystems RCWTs were correlated with plant productivity and ecosystem functions [14,15]. We, therefore, anticipated that the traits of whole root communities would be useful indicators of land transformation.

Here, we asked whether transformation of tropical rain forest into intensive rubber or oil palm mono-plantations would affect functional traits of the root communities. An important functional trait indicating resource conservation is the chemical composition of roots. In our study we determined the concentrations of nutrients and other elements (C, N, P, N, K, S, Ca, Mg, Mn, Fe, Al, Na) in roots from different land use systems. We further measured traits related to plant performance and life style such as root mass, root vitality and colonization with mycorrhizas (EM colonization, AM colonization including vesicles, arbuscules and spores). All traits were determined in mixtures of roots collected in defined soil volumes and therefore represent RCWTs. Specifically, we hypothesized that chemical and performance parameters of root communities vary with forest transformation and are related to transformation intensity. Because land transformation results in degradation of ecosystem functions, we further tested whether RCWTs were correlated with ecosystem properties such as soil carbon and nitrogen concentrations. To test our hypotheses we selected four forest types (oil palm plantations, rubber monoculture, rubber jungle and rain forest) in two landscapes on Sumatra and investigated RCWTs and indicators for ecosystems functions (soil carbon and nitrogen concentrations, leaf litter carbon and nitrogen concentrations, soil available phosphorus and base cations concentrations, soil pH).

## Materials and Methods

### Site description

**Fig 1:**
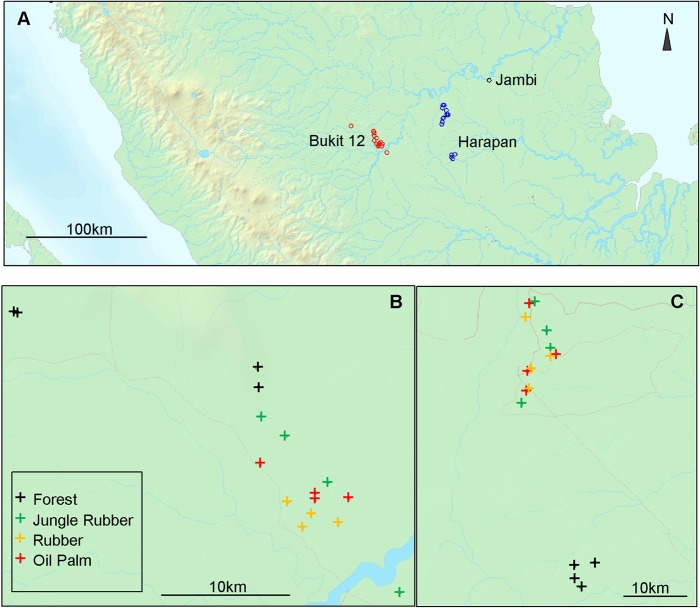
Maps of the province Jambi (A) with the landscapes Bukit 12 (B) and Harapan (C) on Sumatra (Indonesia). The locations of the research plots are indicated.

The study sites were located on Sumatra, Province of Jambi (Indonesia) in two landscapes, i.e., the area of Harapan Rainforest and the area of the National Park Bukit 12 (Fig 1). In each landscape four land use systems were selected: secondary rain forest, jungle rubber, rubber plantations and oil palm plantations. The study areas were in the lowlands (below 100m a.s.l.) on deep, well drained, acid soil with low fertility [6]. The soils are classified as loam acrisol in the Harapan and clay acrisol in the Bukit 12 landscape. The climate is tropical with annual precipitation > 2000mm and only two months with less than 100 mm rain fall. In the Harapan area the annual mean temperature is 26.9°C and the annual precipitation 2332mm (location: Dusun Baru, http://en.climate-data.org/location/595657/); in the Bukit 12 area the mean annual temperature is 26.8°C and the precipitation sum is 2860mm (location: Lubuk Kepayang, http://en.climate-data.org/location/587840/).

### Sampling and export permission

Research permit (Kartu Izin Peneliti Asing, permission number: 333/SIP/FRP/SM/IX/2012) was issued by the Ministry of Research and Technology RISTEK (Kementrian Ristek dan Teknologi, Jakarta, Indonesia). The Research Center for Biology of the Indonesian Institute of Science LIPI (Lembaga Ilmu Pengetahuan Indonesia, Jakarta, Indonesia) recommended issuing a sample collection permit (Rekomendasi Ijin Pengambilan dan Angkut (SAT-DN) Sampel Tanah dan Akar, number: 2696/IPH.1/KS:02/XI/2012). Collection permit (number: S.16/KKH-2/2013) and export permit (reference number: 48/KKH-5/TRP/2014) were issued by the Directorate General of Forest Protection and Nature Conservation PHKA (Perlindungan Hutan dan Konservasi Alam, Jakarta, Indonesia) under the Ministry of Forestry of the Republic of Indonesia. The Chamber of Agriculture of Lower Saxony (Plant Protection Office, Hannover, Germany) issued the import permits (Letter of Authority, numbers: DE-NI-12- 69 -2008-61-EC, DE-NI-14- 08 -2008-61-EC).

### Sampling design

In each of the two landscapes and in each forest type four plots (50 m x 50 m) were installed resulting in 32 sampling sites (Table 1). Oil palm, rubber plantations and rubber jungle were sampled in October and November 2012 and rain forest in November and December 2013. In each plot, subplots of 5m x 5m were defined and soil samples were collected in three of these subplots (designated as a, b, c). In each subplot five soil cores (0.04 m diameter and 0.20 m depth) were extracted (four towards the corners and one in the centre of the subplot) at a distance of more than 1 m. Leaf litter was removed before soil sampling and kept separately. In total 480 soil cores were taken in both landscapes (2 landscapes x 16 plots x 3 subplots x 5 soil cores). Soil cores and litter samples were stored individually in plastic bags in cool bags and transported to the University of Jambi, where they were stored at 4°C until processing.

**Table 1:** Geographic location of the research plots in two landscapes and four forest types on Sumatra (Indonesia).

	Bukit 12				Harapan		
Plot	latitude	longitude	altitude (m asl)	plot	latitude	longitude	altitude (m asl)
BF1	S 01°59'42.5''	E 102°45'08.1''	83	HF1	S 02°09'09.9''	E 103°21'43.2''	76
BF2	S 01° 58'55.1''	E 102°45'02.7''	77	HF2	S 02°09'29.4''	E 103°20'01.5''	75
BF3	S 01°56’33.9''	E 102°34’52.7''	87	HF3	S 02°10'30.1''	E 103°19'57.8''	58
BF4	S 01°56’31.0''	E 102°34’50.3''	87	HF4	S 02°11'15.2''	E 103°20'33.4''	77
BJ1	S 02°08'25.6''	E 102°51'04.3''	74	HJ1	S 01°55'40.0''	E 103°15'33.8''	51
BJ2	S 02°01'49.7''	E 102°46'16.7''	76	HJ2	S 01°49'31.9''	E 103°17'39.2''	84
BJ3	S 02°03'46.7''	E 102°48'03.5''	89	HJ3	S 01°50'56.9''	E 103°17'59.9''	95
BJ4	S 02°00'57.3''	E 102°45'12.3''	60	HJ4	S 01°47'07.3''	E 103°16'36.9''	57
BR1	S 02°05'30.7''	E 102°48'30.7''	71	HR1	S 01°54'39.5''	E 103°16'00.1''	77
BR2	S 02°05'06.8''	E 102°47'20.7''	95	HR2	S 01°52'44.5''	E 103°16'28.4''	59
BR3	S 02°05'43.0''	E 102°46'59.6''	90	HR3	S 01°51'34.8''	E 103°18'02.1''	90
BR4	S 02°04'36.1''	E 102°46'22.3''	51	HR4	S 01°48'18.2''	E103°15'52.0''	71
BO1	S 02°04'26.1''	E 102°48'55.1''	75	HO1	S 01°54'35.6''	E 103°15'58.3''	81
BO2	S 02°04'32.0''	E 102°47'30.7''	84	HO2	S 01°53'00.7''	E 103°16'03.6''	55
BO3	S 02°04'15.2''	E 102°47'30.6''	71	HO3	S 01°51'28.4''	E 103°18'27.4''	64
BO4	S 02°03'01.5''	E 102°45'12.1''	34	HO4	S 01°47'12.7''	E 103°16'14.0''	48

O = oil palm plantation, R = rubber plantation, J = jungle rubber, F = secondary rain forest.

### Sample preparation

Each soil core was weighed, sieved subsequently through two sieves with 10 and 5 mm mesh size and separated into roots and bulk soil. The five samples from the same subplot were pooled and well mixed yielding one root and one bulk soil sample per subplot. Litter samples of a subplot were also pooled yielding a total number of 96 pooled samples per fraction.

Litter samples were dried in an oven at 80°C for 48h. Fresh bulk soil samples (about 20 g) were initially air dried and then oven dried (105°C for 48 h) to determine the soil water content according to the following equation:

$$\text{Relative soil water content}\;\left({\text{g g}}^{-1}\;\text{soil}\right)\;=\;\left(\frac{\text{weight of fresh soil}\;\left(\text{g}\right)-\text{weight of oven dried soil}\;\left(\text{g}\right)}{\text{weight of fresh soil}\;\left(\text{g}\right)}\right)$$

Pooled root samples were washed and patted dry with tissue paper. The fresh root mass of the sample was weighed. The roots were separated into coarse and fine roots according to the root diameter. Fine roots (diameter ≤ 2 mm) were weighed, stored in wet tissue paper at 4°C, used for root vitality and mycorrhizal analysis, and were subsequently oven-dried at 60°C for 48h. Fine root dry mass was calculated as:

$$\text{Fine root mass}\;\left({\text{g kg}}^{-1}{\text{soil}}_{\text{dw}}\right)\;=\;\left(\frac{\text{dry weight of fine roots of subplot a}+\text{subplot b}+\text{subplot c}\;\left(\text{g}\right)}{\text{dry weight of soil of subplot a}+\text{subplot b}+\text{subplot c}\;\left(\text{kg}\right)}\right)$$

Dry aliquots of soil, roots and litter were stored in 50 ml reaction tubes (Falcon tube 50 ml, 115 x 28 mm, Sarstedt, Nümbrecht, Germany). Before closing the screw cap, a small reaction tube (Eppendorf micro tube, 1.5 ml, Sarstedt, Nümbrecht, Germany) with perforated walls containing silica gel (10 g (40 x 90 mm) desiccant bag silica gel orange, Carl Roth, Karlsruhe, Germany) was added. The samples were shipped to the University of Göttingen (Göttingen, Germany), IPB Bogor Agricultural University (Bogor, Indonesia) and Tadulako University (Palu, Indonesia) for further analysis.

### Analysis of root vitality and ectomycorrhizal (EM) colonization

The root tips of fresh fine roots were inspected using a dissecting microscope with an integrated camera (Leica EZ4HD, Wetzlar, Germany) at 35-fold magnification. Aliquots of fine roots were placed in a water-filled Petri dish (Petri dish 92 x 16 mm, Sarstedt, Nümbrecht, Germany). In general, 250 roots tips were counted and scored as vital and dead root tips after colour of vascular tissue, strength and flexibility as described by Allen et al. (2000). On the vital root tips the number of EM root tips was counted. EM root tips were recognized by presence of a sheath or mantle of fungal tissue which enclosed the root and emanating hyphae [18]. Dead, non-EM, and vital EM root tips were documented by photos taken with the microscope camera.

### Arbuscular mycorrhizal (AM) colonization

Up to 25 fine root fragments per subplot with a length of 20 to 30 mm measured from the root tip were stored in reaction tubes (Eppendorf micro tube 2ml, Sarstedt, Nümbrecht, Germany) containing 70% ethanol (Rotisolv HPLC Gradient, Carl Roth, Karlsruhe, Germany). Roots were stained following the method of Vierheilig *et al*. [22]. The root segments were washed several times with ultra-purified water (ultra-pure water system, Arium 611, Sartorius, Göttingen, Germany), briefly surfaced-dried on tissue paper and then bleached in 2 ml of 10% potassium hydroxide (KOH, Merck, Darmstadt, Germany) for 90 min at 90°C. Because not all roots were bleached after one KOH treatment, this step was repeated with variation of the incubation time and temperature until the objective was achieved. The bleached roots were carefully washed up to three times with ultra-purified water to remove the KOH and then stained in 2 ml of a vinegar-ink-solution (10% acetic acid (Merck, Darmstadt, Germany), black ink (Sheaffer Skrip, Shelton, USA) and ultra-purified water with a ratio of 1:1:8 for 45 min at room temperature. The stained roots were washed with ultra-purified water to remove superfluous dye. Roots were preserved up to eight weeks in lactoglycerol consisting of 86% glycerol (Carl Roth, Karlsruhe, Germany), 80% lactic acid (Carl Roth, Karlsruhe, Germany) and ultra-purified water with a ratio of 1:1:1 before preparing microscope object slides.

For microscopic analysis, roots were cut into small segments (10 mm) and arranged with forceps in a drop of lactoglycerol as the mountant on a microscope object slide. Cover slides were gently pressed on root segments and flattened overnight using a lead weight (weight between 40 and 50 g). Subsequently, the cover slides were sealed with colorless nail polish to protect the specimen from drying. Three slides per sample were prepared and analyzed.

The gridline intersection method after McGonigle *et al*. [23] was used to determine AM colonization. The slides were placed under a compound microscope (Axio Observer Z.1, Zeiss, Jena, Germany). With the computer program AxioVision LE (Zeiss, Jena, Germany) a gridline was generated on the considered section (magnification 400x, distance between the intersects 100 μm) and the presence or absence of the following structures was recorded in 120 intersects per sample: AM hyphae, arbuscules, and vesicles. For each recorded arbuscule and vesicle, a hypha was also counted because these structures are always co-occurring. For each sample 120 intersects were counted. AM colonization was calculated as:

$$\text{AM colonization}\;\left(\%\right)=\frac{\text{number of hyphae}}{\text{total number of intersects}}\;*\;100$$

The relative abundance of arbuscules and vesicles was calculated correspondingly.

### Determination of arbuscular mycorrhizal spore abundance

Air dried samples of bulk soil were stored in sealed plastic bags at 4°C. Spores from each soil sample (n = 480) were isolated as described by Gerdemann and Nicolson [24]. Twenty gram of soil of each sample was suspended in 500 ml of water, stirred manually for 10 min. The suspension was passed through sieves, which were arranged in a descending order from 250 μm, 125 μm and 63 μm and washed with tap water. The material retained on the sieves were layered onto a a water-sucrose solution (50%) gradient and centrifuged at 900 x g for 2 min [25]. The supernatant was washed with tap water for 3 min in a 63 μm sieve, filtrated onto a gridded filter paper, then placed in a 90 mm diameter Petri dish. The spores obtained from all sieves were counted under a binocular stereomicroscope with 100 to 400-fold magnification (Olympus SZ61, Osaka, Japan). The number of spores were expressed as spores per 20 g soil sample.

### Element analyses in plant and soil fractions

Dry samples of soil, roots and litter were ground to a fine powder in a ball mill (MM 2000, Retsch, Haan, Germany). Aliquots of 0.7 to 0.9 mg per sample were weighed into tin capsules (5 x 9mm, HEKAtech, Wegberg, Germany) and used for carbon and nitrogen analyses in an Elemental Analyzer (EA 1108, Carlo Erba Instruments, Milan, Italy). Acetanilide (C: 71.09%, N: 10.36%, HEKAtech, Wegberg, Germany) was used as the standard.

For analyses of the elements Al, Ca, Fe, K, Mg, Mn, Na, P and S (aluminum, calcium, iron, potassium, magnesium, manganese, sodium, phosphorus and sulfur) a milled aliquot of 50 mg of dry soil or fine roots of each sample was digested in 2 ml of 65% nitric acid (HNO_3_, Merck, Darmstadt, Germany) for 14h at 200°C. Afterwards each extract was completely transferred into an Erlenmeyer flask. The polytetrafluoroethylene tubes (Loftfields Analytische Lösung, Neu Eichenberg, Germany) used for the extraction were washed with HPLC grade water (Chromanorm, VWR, Darmstadt, Germany), the washing solution was filtered through black ribbon filter paper (filter papers MN 640w, ᴓ 90mm, ashless, Macherey-Nagel, Düren, Germany) into the Erlenmeyer flask and the volume was adjusted to 25 ml with HPLC grade water. Then elements in the extract were analysed by inductively coupled plasma optical emission spectrometry (ICP OES, iCAP 6300 Series, Thermo Fischer Scientific, Dreieich, Germany).

$$\text{Element concentration}\;\left({\text{mg g}}^{-1}\right)=\frac{\text{element concentration}\;\left({\text{mg l}}^{-1}\right)\;\text{x volume}\;\left(\text{l}\right)}{\text{mass of dry material}\;\left(\text{g}\right)}$$

To calculate the sum of base cations, the concentrations of potassium, magnesium and calcium were converted from mg g^-1^ into μmol g^-1^ and then added.

For the extraction of available phosphorus in soil the method of Bray and Kurtz [26] was used. Air dried soil samples were sieved through a 2 mm mesh. Two grams of soil from each sample were mixed with 15 ml of Bray solution containing 0.03 N NH_4_F and 0.025 N HCl and were shaken (Finofors AG, Basel, Switzerland) for 5 min at 180 rpm at room temperature. After shaking, the suspensions were filtered through a phosphorus-free folded filter (filter papers MN 280 ¼ 125mm, Macherey-Nagel, Düren, Germany). Phosphorus concentrations of the filtrates were analysed by ICP OES (iCAP 6300 Series, Thermo Fischer Scientific, Dreieich, Germany).

### Determination of soil pH

Soil pH was determined at a depth of 0.01 m. Soil was mixed with deionized water (1:4) and used for pH measurements.

### Maps of the sampling site

Maps of plot locations were generated the free software package GPS Visualizer (http://www.gpsvisualizer.com/) [27].

### Data analysis

The samples of each subplot (3 per plot) were analyzed individually. In rare cases (4 of 96 only 1 or 2 samples per subplot) were available. The subplot data were used to calculate plot means. All further analyses were based on plot means. Plots means were used as input parameters to construct the data matrices for principle component analysis (PCA). Significant principle components (PCs) were determined by broken stick analysis. Non-metric multidimensional scaling (NMDS) was conducted with Gover as similarity measure. Multivariate analyses were conducted with the PAST free software package 2.17c (http://folk.uio.no/ohammer/past/, [28]). The data were subjected to test the requirement of normal distribution by the Shapiro Wilks test (P ≥ 0.05). When the P value of the Shapiro Wilks test was < 0.05, data were ln- or (-1/square-root)-transformed to achieve normal distribution. In one case (ectomycorrhizal colonization), it was not possible to satisfy this criterion. The data were nevertheless included, but their in- or exclusion did not affect the final result. Because the data had different units and were subjected to different transformation procedures, the resulting matrix was z-score normalized and then used for the analyses. Because of the use of normalized data, the relative importance of individual factors was not considered, but their correlation coefficient R^2^ with the PCs. A linear mixed model with landscape a fixed factor and land use system as random factor nested in landscape was used to test the contribution of the variables land use system and landscape to the PCs (Statgraphics, Centurion XV, St Louis, Mo, USA). Variance component analysis revealed no contribution of the factor landscape on PC1. Therefore, one-way ANOVA with the only factor land use system was conducted for the PC1 data (post hoc test: Tukey HSD) and the data were used to develop a general linear model with PC1 as the dependent variable and soil and litter properties as independent variables. The categorical factors land use system and landscape were not included in the model because they had been used to develop PC1. Combinations of all eight predictors variables (soil N, soil C, soil pH, soil P, soil cations, soil water content, litter C, litter N) were tested and the model with the lowest Akaike information criterion (AIC = 0.569) containing three variables was chosen. When the data were not-normal distributed the Kruskal Wallis test was conducted and medians and range of the data were indicated.

### Data deposition and availability

The raw data of this study are deposited and available in the Dryad repository under doi:10.5061/dryad.qf362

## Results

### Root community-weighed traits are massively affected by the land use system

**Fig 2:**
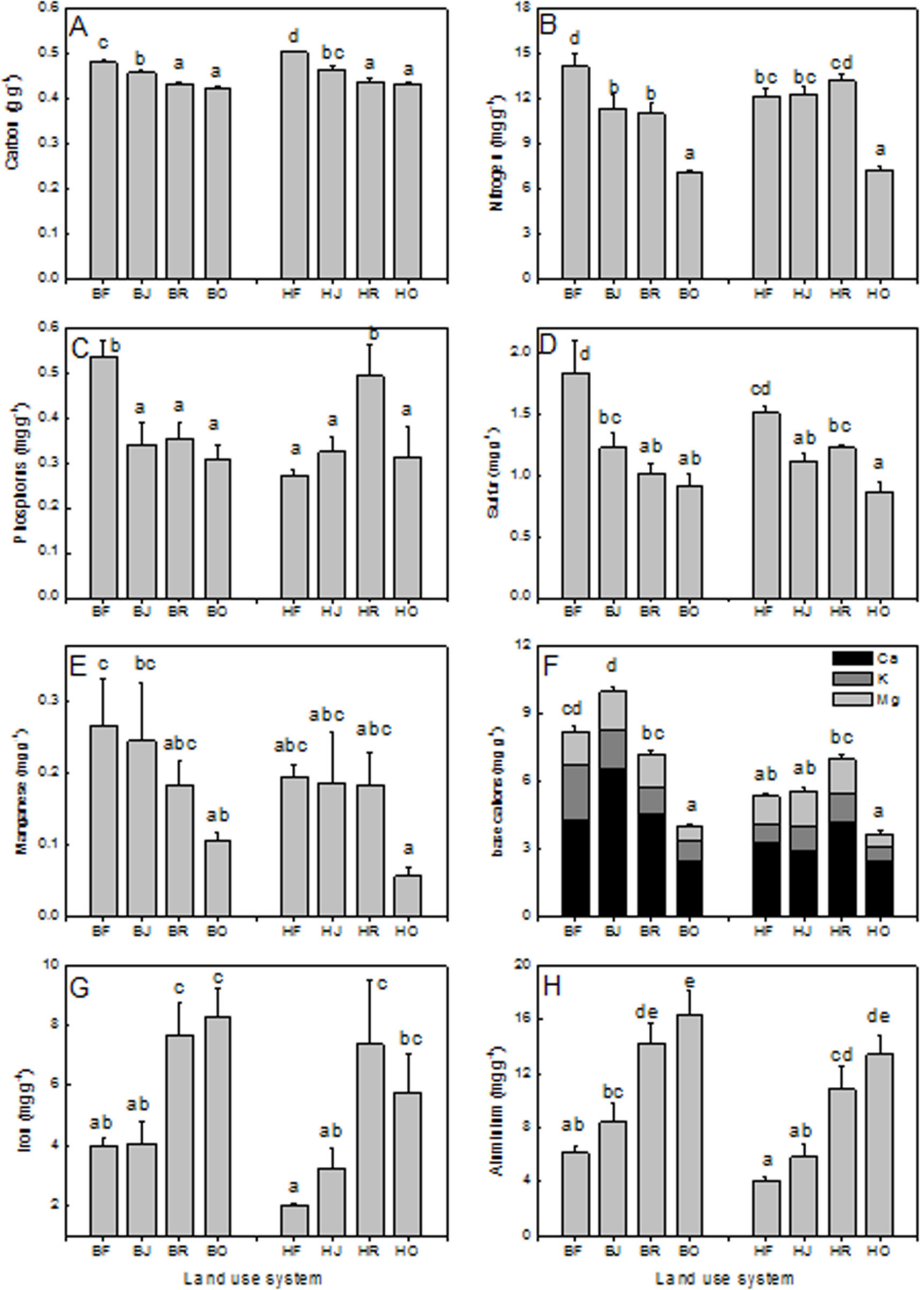
Chemical composition of roots in different land use systems. Carbon (A), nitrogen (B), phosphorus (C), sulfur (D), manganese (E), base cations (F), iron (G) and aluminium (H) determined as root community-weight traits. Data indicate means (± SE). Different letters indicate significant differences at P < 0.05. B = Bukit 12, H = Harapan, O = oil palm, R = rubber plantation, J = jungle rubber, F = forest.

Our measurements of the root nutrient elements represent RCWTs because the roots were collected in defined soil volumes representing mixtures of tree species and understory weeds on the plots. Root carbon, nitrogen, sulfur, manganese, and base cations concentrations showed a decline in rubber and oil palm plantations compared with those from forest systems (Fig 2A, 2B, 2D, 2E and 2F). In both landscapes, Harapan and Bukit 12, the decline in the root nutrient concentrations with land use type was similar. No clear influence of the land use system was observed on the root phosphorus concentrations (Fig 2C). The concentrations iron and aluminium, both potentially toxic compounds at high concentrations, showed strong increases in roots of oil palm and rubber plantations compared to jungle rubber and rain forest roots (Fig 2G and 2H).

**Fig 3:**
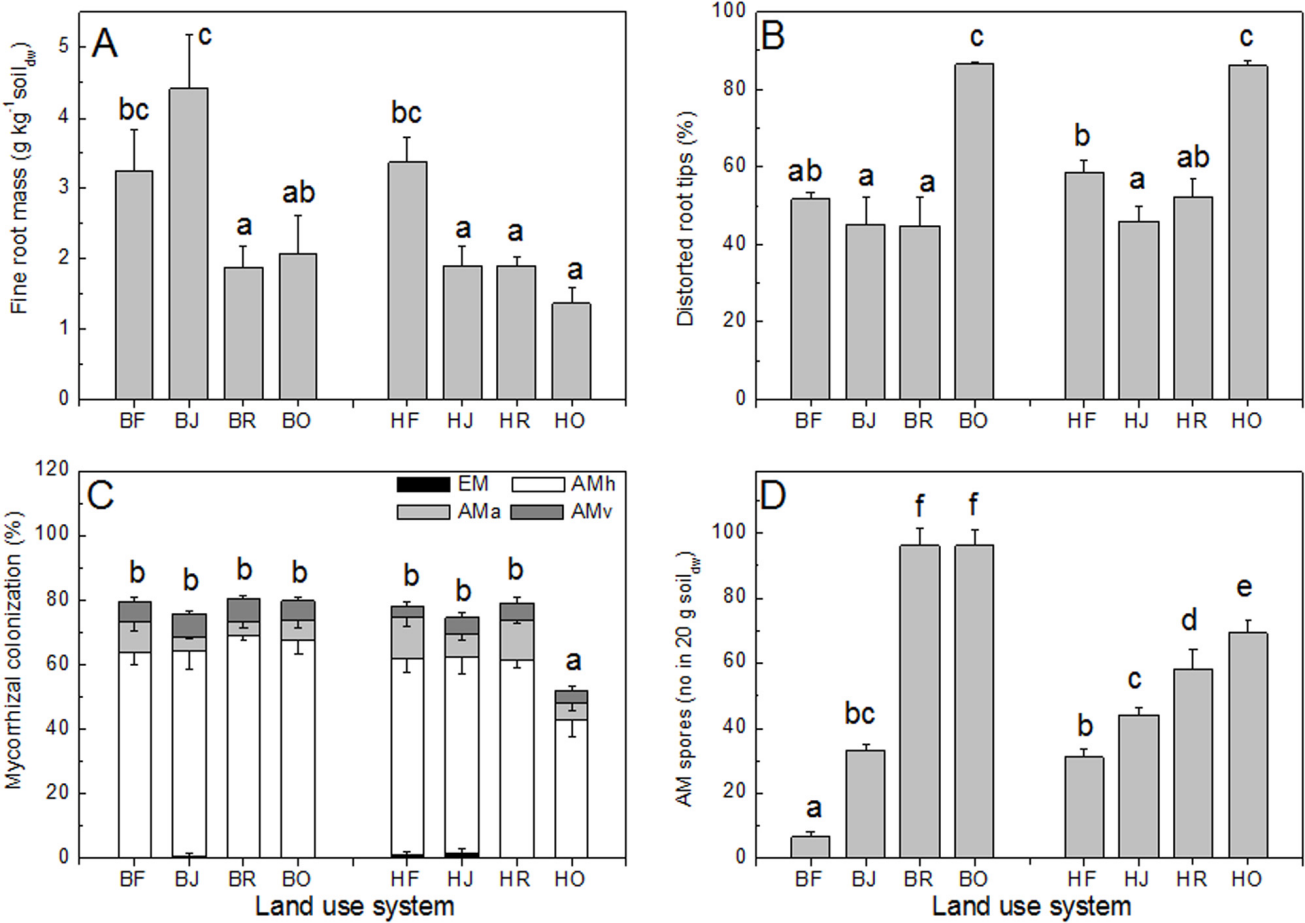
Performance parameters of roots in different land use systems. (A) Fine root mass to a depth of 0.2 m, (B) Fraction of distorted root tips (100% is the total number of inspected root tips), (C) Fraction of the inspected root lengths colonized with mycorrhizal hyphae (AMh), arbuscules (AMa), vesicles (AMv) and fraction of vital root tips colonized with ectomycorrhizas (EM), (D) Number of arbuscular mycorrhizal spores. Data indicate means (± SE). Different letters indicate significant differences at P < 0.05. B = Bukit 12, H = Harapan, O = oil palm, R = rubber plantation, J = jungle rubber, F = forest.

We further determined RCWTs that are related to root vitality and mycorrhizal association (fine root mass, colonization by ectomycorrhizal and AM fungi, AM vesicles, AM arbuscles, AM spores in soil, dead root tips) (Fig 3). Fine root mass was higher in rain forest than in oil palm plots, where also the highest fraction of distorted root tips was found (Fig 3A and 3B). The fraction of mycorrhizal roots was stable (74.4 ± 1.7%) with the exception of the oil plantations in Harapan (51.8 ± 7.5%, Fig 3C). EM colonization was detected in some plots in Harapan rain forest with a maximum of 6% in one plot and in jungle rubber in both landscapes, but their overall abundances were rare (Fig 3C). AM spore abundance was lowest in the rain forest and highest in oil palm plantations (Fig 3D).

### Root community-weighed traits indicate transformation intensity

**Fig 4:**
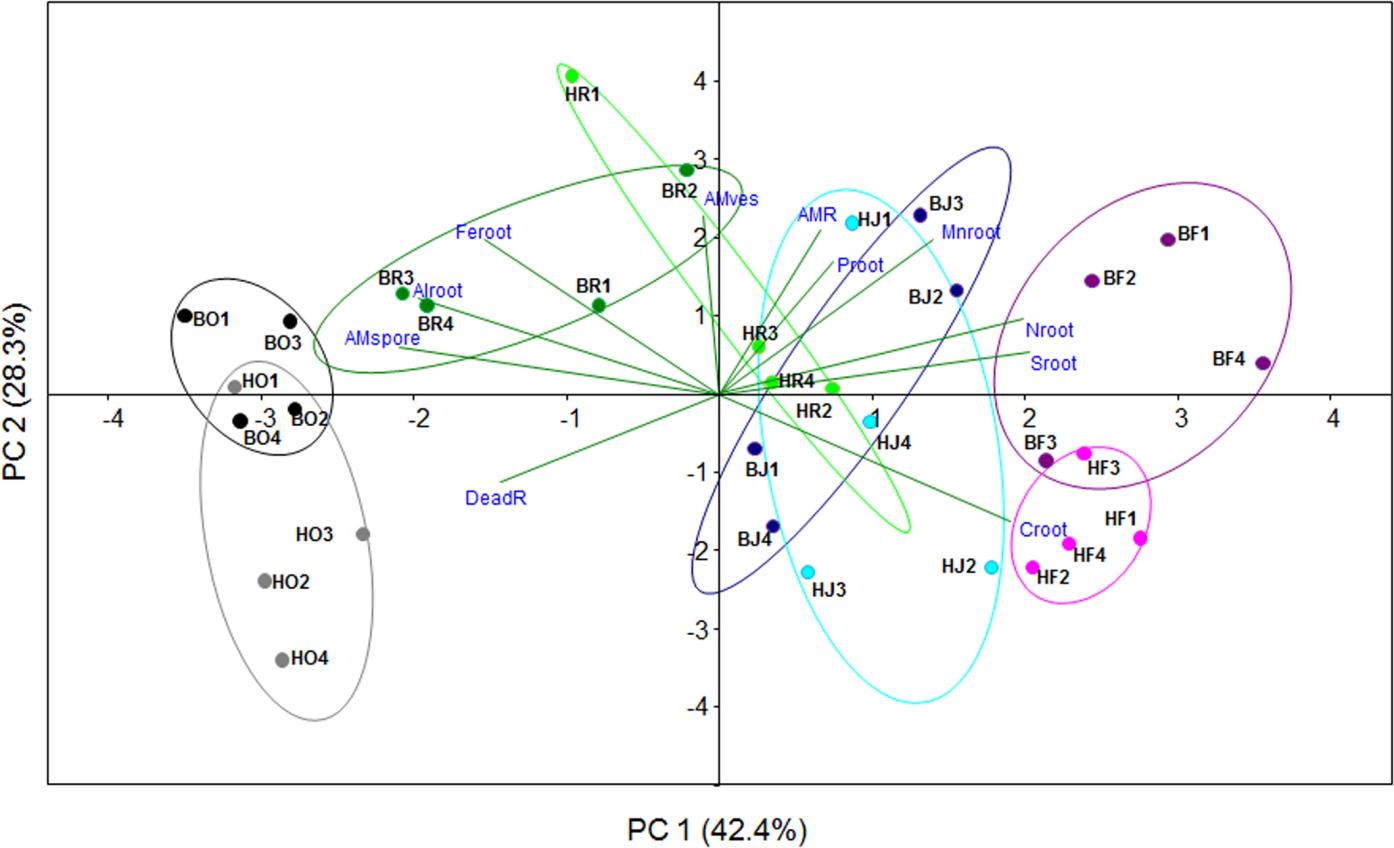
Principle component analysis of root community-weighed traits. The traits used for PCA and their abbreviations are listed in Table 2. B = Bukit 12, H = Harapan, O = oil palm, R = rubber plantation, J = jungle rubber, F = forest.

PCA with all sixteen RCWTs shown in Fig 2 and Fig 3 revealed that the variables ectomycorrhizal colonization, abundance of AM arbuscules and Na resulted in insignificant loadings with R < 0.5 and the parameters fine root mass and base cations showed collinearity with other root properties and were therefore removed. The reduced PCA was based on eleven RCTWs (Table 2) and resulted in two significant PCs that explained 42.4% (PC1) and 28.3% (PC2) of the variation, respectively (Fig 4). PC1 separated the land use systems with the rain forests exhibiting the most positive and oil palm plantations the most negative scores (Fig 4). Positive PC1 loadings with correlations of R ≥ 0.5 were C, N, S, and Mn (Table 2). Negative PC1 loadings with R ≤ -0.5 were AM spores, dead root tips, Al and Fe (Table 2). RCWTs related to mycorrhization (AM colonization, AM vesicles) and to phosphorus were not strongly correlated with PC1 (Fig 4, Table 2), but were significant loadings on PC2.

**Table 2:** PCA loadings for correlations of root traits with PC1 and PC2.

Trait name	Abbreviation	PC1	PC2
Sulfur	Sroot	0.838	0.180
Nitrogen	Nroot	0.821	0.326
Carbon	Croot	0.786	0.551
Manganese	Mnroot	0.579	0.670
AM root colonization	AMR	0.275	0.709
Phosphorus	Proot	0.306	0.571
AM vesicles	AMves	-0.045	0.773
Iron	Feroot	-0.634	0.665
Dead root tips	DeadR	-0.592	-0.381
Aluminium	Alroot	-0.817	0.414
AM spores in soil	AMspore	-0.866	0.200

To quantify the influence of the factors landscape and land use systems on the variation of the PC1 and PC2 scores, the data were analyzed by general linear mixed models. Significant models were obtained for both PCs with R^2^_(adjusted for df)_ explaining 92.6% of the variation of the PC1 scores and 32.9% of the PC2 scores (Table 3). However, the only significant factor was land use system (Table 3). Analyses of the variance components (in the order of nesting) showed that landscape contributed 0%, land use system 94.1% and the error term 5.9% to the variation of PC1. For PC2 the contributions of the components to the total variation were error term (58.4%), landscape (23.1%) and land use system (18.5%). Mean values of the PC1 scores ordered the land use systems according to transformation intensity in the order: forest > rubber jungle > rubber > oil palm (Table 4).

**Table 3:** General linear mixed model for PC1 and PC2 as dependent variables and landscape and land use system (LUS) as categorical factors.

Source	Sum of Squares	Df	Sum of Squares	F-Ratio	P-Value
**Analysis of Variance for PC21**					
Model	136.46	7	19.49	56.31	<0.001
Residual	8.31	24	0.35		
landscape	0.92	1	0.92	0.04	0.847
LUS(landscape)	135.54	6	22.59	65.25	<0.001
Residual	8.31	24	0.35		
Total (corrected)	144.77	31			
**Analysis of Variance for PC2**					
Model	46.45	7	6.63	3.17	0.016
Residual	50.18	24	2.09		
landscape	17.97	1	17.97	3.79	0.100
LUS(landscape)	28.47	6	4.74	2.27	0.071
Residual	50.18	24	2.09		
Total (corrected)	96.63	31			

Landscape was set as fixed and LUS as random factor nested in landscape.

**Table 4:** Mean PC scores of the land use systems.

Site	PC1 ± SE	PC2 ± SE
BF	2.77 *±* 0.31e	0.74 ± 0.62ab
HF	2.34 ± 0.17de	-1.68 ± 0.32ab
BJ	0.86 ± 0.33c	0.30 ± 0.19ab
HJ	1.05 ± 0.26cd	-0.66 ± 1.05ab
BR	-1.25 ± 0.45b	1.60 ± 0.42b
HR	0.09 ± 0.37bc	1.22 ± 0.95ab
BO	-3.06 ± 0.17a	0.35 ± 0.36ab
HO	-2.84 ± 0.18a	-1.87 ± 0.74a

Different letters in columns indicate significant differences at P < 0.05 determined with the HSD test. B = Bukit 12, H = Harapan, O = oil palm, R = rubber plantation, J = jungle rubber, F = forest.

### Transformation intensity is linked with ecosystem properties

**Fig 5:**
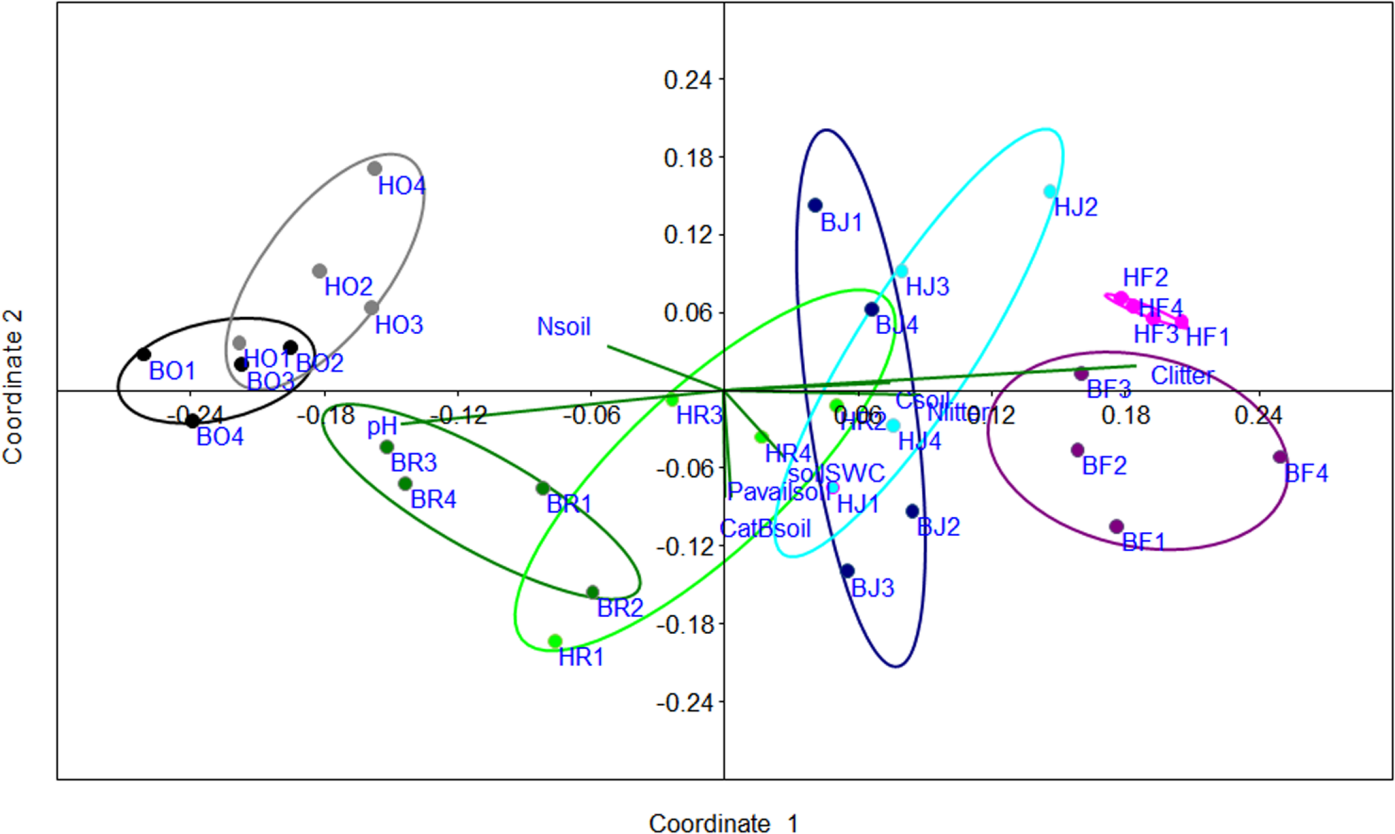
Non metric multidimensional scaling (NMDS) of root community-weighed traits. RCWTs with R>0.5 and R< 0.5 from Table 1 for PC1 were used for NMDS. The following environmental parameters were plotted as explanatory variables: nitrogen and carbon concentrations in soil and litter (Nsoil, Csoil, Clitter, Nlitter), available phosphorus in soil (Pavailsoil), sum of basic cations in soil (CatBsoil), soil water content (soilSWC) and soil pH (pH). B = Bukit 12, H = Harapan, O = oil palm, R = rubber plantation, J = jungle rubber, F = forest. Stress: 0.106, R^2^ for coordinate 1: 0.785 and for coordinate 2: 0.0735.

In tropical ecosystems loss of forest cover and conversion into agricultural land use systems has often been linked with loss in soil fertility and soil carbon contents [9,11]. We, therefore, asked whether the RCWTs that ordered the land use systems according to transformation intensity also corresponded to loss of ecosystem functions indicated by soil properties. Soil (sum of base cations, available phosphorus, pH, water content, carbon, nitrogen) and litter properties (carbon, nitrogen), which we measured as proxies for ecosystem functions showed significant variations among different sites (Table 5). An NMDS conducted with the significant loadings of RCWTs for PC1 (Table 2) and the environmental variables (Table 5) as explanatory vectors indicated that soil pH and soil N were related to the negative scores of oil palm and rubber plantations, while soil C and litter N and C were related to the positive scores of rain forest and jungle rubber (Fig 5). However, it should be noted that the overall pH differences between the plots were small (Table 5, mean pH of rain forest plots: 4.25 ± 0.03 and mean pH of the other land use systems: 4.46 ± 0.13, P = 0.002).

**Table 5:** Median and range of environmental properties.

Plot	C	Range	N	Range	base cations	Range	P	Range	Water	Range	pH	Range	C	Range	N	Range
	(g g^-1^ soil_dw_)		(mg g^-1^ soil_dw_)		g^-1^ soil_dw_)		(mg g^-1^ soil_dw_)		(mg g^-1^ soil_fw_)				(mg g^-1^ soil_dw_)		(mg g^-1^ soil_dw_)	
BF	0.29	0.26	2.06	2.06	3.04	10.05	0.0048	0.0067	0.29	0.26	4.2	0.2	424.2	97	14.22	6.06
BJ	0.36	0.18	3.37	0.94	5.96	2.2	0.0019	0.0016	0.36	0.18	4.5	0.2	454	120.8	12.4	3.07
BR	0.25	0.17	1.27	2.18	1.39	2.69	0.0012	0.0006	0.25	0.17	4.5	0.2	377.3	115	11.48	5.77
BO	0.29	0.09	1.84	1.7	2.12	3.41	0.0043	0.0112	0.29	0.09	4.45	0.2	347.3	100.4	12.11	6.54
HF	0.25	0.05	1.47	0.49	1.98	0.72	0.0021	0.0012	0.25	0.05	4.3	0.2	469.8	46.3	13.24	1.37
HJ	0.22	0.09	1.59	1.14	0.79	5.42	0.0022	0.0133	0.22	0.09	4.3	0.2	458.7	38	14.64	3.21
HR	0.24	0.05	1.45	0.56	3.52	3.61	0.0019	0.0032	0.24	0.05	4.45	0.5	435.8	76.9	15.06	3.42
HO	0.2	0.1	1.09	1.06	1.47	4.25	0.0038	0.016	0.2	0.1	4.5	0.3	362.2	46.4	12.58	1.4
Test statistic	18.93		14.64		10.88		15.39		12.84		19.8		16.84		10.31	
P value	**0.008**		**0.041**		0.144		**0.031**		0.076		**0.01**		**0.018**		0.172	

P values of the Kruskal Wallis rank test for the land use systems are indicated. B = Bukit 12, H = Harapan, O = oil palm, R = rubber plantation, J = jungle rubber, F = forest.

To find out whether the PC1 scores which distinguish the land use systems independently from landscape can be quantitatively related to ecosystem functions, we tested general linear models. The PC1 scores were used as dependent and the environmental properties as independent variables. The categorical factors land use system and landscape were not included in the model, because they had been used to determine the PC1 components. The model with the lowest AIC contained three significant components: soil nitrogen concentration, soil pH and litter carbon concentration (Table 6). The model explained 70% (R^2^ adjusted for d.f.) of the variation. The *P*-value of the Durbin-Watson statistic was > 0.05 and therefore the model was not significantly affected by serial autocorrelation in the residuals.

**Table 6:** Best general linear model for the relationship of PC1 with ecosystem properties.

Source	Sum of Squares	Df	Mean Square	F-Ratio	P-Value
Model	106.2	3	35.4	25.71	<0.0001
Residual	38.55	28	1.37		
Nsoil	6.37	1	52.15	37.88	0.0401
pH	26.14	1	26.14	18.99	0.0002
Clitter	52.16	1	52.16	37.88	<0.0001
Residual	38.55	28	1.37		
Total (corrected)	144.76	31			

## Discussion

### Root community-weighed traits and soil properties vary with forest transformation

Recent studies highlight the importance of functional structures of communities rather than their biodiversity for ecosystem functioning [29–31]. Our study clearly demonstrates a decline of positive RCWTs such as high root mass and high nutrient concentrations in mono-culture oil palm plantations compared with rain forest. Based on our design we cannot distinguish whether the enhanced properties of the root communities in the rain forest were the result of tree phylogenic diversity or of trait-enrichment due to the presence of forest tree species with distinct features. We expected that the impact of dominant trees might have been traced by an effect of the associated EM on RCWTs, because the root nutrient status of forest trees is affected by symbioses with AM or EM fungi and fungal species identities [32–34]. However, our data did not reveal an influence of the land use system on the mycorrhizal life traits. In contrast to the relatively stable AM colonization, AM spore abundance varied strongly with transformation system. Fungi are propagated by spores, but spores are also resting structures, by which the fungi survive unfavorable conditions [35]. In tropical systems increased spore abundance correlated with decreased soil fertility [36]. The increased AM spore abundance in oil palm and rubber monocultures, thus, points to links of these agricultural systems with ecologically important life traits.

A negative impact of monoculture oil palms was evident on soil carbon and nitrogen contents. Conversion of tropical forests into agricultural production systems has often been shown to result in decreased soil carbon and nitrogen pools [37–41]. The magnitude of this effect in our study was similar to that in other tropical transformation system, e.g. in cash crops such as maize on Central Sulawesi (Indonesia) [9]. In comparison with agricultural land use, agro-forestry systems recovered soil fertility [9]. A beneficial effect of jungle rubber, an extensive agro-forest land use system, on soil properties was confirmed in our study because the carbon and nitrogen concentrations in soil of this system were even higher or, at least, as high as in rain forest soil. This finding is important because soil fertility has direct consequences for ecosystem services such as biomass production, carbon cycling and carbon sequestration and has been identified as the major regulator of forest carbon balance [42].

Soil properties and vegetation mutually influence each other because both compartments are connected by matter flux. Alterations in plants traits are transmitted to the soil by the input of degrading leaf and root litter as well as by root physiological activities (exudation of carbohydrates, organic acids, nutrient uptake) [43,44]. Therefore, RCWTs and soil properties are to some extent inter-dependent. Our study provides some insights into the nature of these links because the RCWTs that reflected transformation intensity were also linked with soil and litter properties, i.e., soil pH, soil N and litter C concentrations. This finding is interesting because litter carbon is the result of litter degradability, which in turn is driven by plant functional traits [45]; soil nitrogen is important for soil fertility and forest productivity and therefore, eventually has strong impact on forest carbon cycling [42]. Our findings, thus, link functional structures of root communities with ecosystem functions, notably with those functions that are more important for carbon sequestration than climate or the rising atmospheric CO_2_ concentration [42,45]. This finding implies that RCWTs could be an important indicator for the functionality of above- and below-ground ecosystem interactions.

Based on the present data, the cause-effect relationships remains unknown because mono-culture species with unfavorable root traits could have affected soil properties or management could have altered soil properties with negative consequences for root traits. Regardless the ultimate reason, our results suggest that the loss in ecosystem functions in mono-cultures was accompanied by complex alterations of root functional traits. Increased transformation intensity was associated with diminished nutrient concentrations and low root mass on the hand and increased concentrations of potentially phytotoxic metals (Al, Fe) and enhanced root tip mortality on the other hand. The transformation intensity was thus indicated by contrasting behavior of distinct RCWTs and not by the loss of traits abundance *per se*. Consequently, we may expect that any management measure that improves root vitality may, eventually, enhance the ecological functions of tropical production systems. It will be important to investigate this suggestion in future studies.

### Degradation of root health is related to accumulation of plant toxic elements

Chemical root traits that distinguished the monocultures, especially the oil palms, from ecosystems with higher tree diversity were the enrichments in Fe and Al. Plant availability of Al is modulated by soil acidity [46]. The soils in the Jambi lowland region are acrisols with pH values of 4.5 and below. In Bukit 12 higher concentrations of exchangeable Al were present than in Harapan (0.54 ± 0.18 mg g^-1^ soil_dw_ versus 0.28 ± 0.04 mg g^-1^ soil_dw_), but without showing a clear gradient among the land use systems as found here for the root communities [47]. In each landscape the exchangeable Fe concentrations were highest in rain forest soil [47], where root communities showed the lowest Fe enrichment. Therefore, the Al and Fe enrichments in roots did not simply reflect soil conditions.

Excess Al accumulation is known to limit plant performance and affects root growth [48–50]. Indeed, the morphological appearance of the oil palm roots on our plots resembled the symptoms of Al toxicity with stubby root systems lacking fine root branches with many brownish, distorted root tips [51]. Although oil palms are often cultivated in acid soils [52] injury due to unfavorable soil conditions cannot be excluded. In field studies, a negative correlation between exchangeable Al in soil and root density of oil palms was found [53]. Controlled studies confirmed the negative impact of Al on oil palm roots, especially on the length of the lateral roots and number of root tips [54]. Cristancho *et al*. [54] further showed that Al-stressed oil palms excreted significant concentrations of oxalic acids. Plant exudation of organic acids influences the availability of other soil elements and mobilizes for example phosphorus and Fe [55,56]. Here, we found high Fe concentrations in roots, whereas soil phosphorus availability was low and root phosphorus concentrations remained unaffected by the land use system. Excess Fe causes oxidative stress leading to cell destruction [56] and may have caused here, together with Al, enhanced root tip mortality. It is important to note that the pH across all forest types was low, but not lower in plantations than in forest soil. Therefore, low pH may be a pre-requisite, but was not the immediate reason for the observed decline in root health.

Currently, we can only speculate about the reasons for root distortion in oil palm plantations. One possibility is that mono-cultures alter the soil microbial flora with negative effects on Al or Fe solubilization and plant availability as found in other countries [57]. AM colonization protects plant roots from Al stress [58], but here variation in AM abundance was unrelated to Al concentrations. Phylogenetic analyses have shown high Al tolerance in tropical forest trees [59–62]. Therefore, it is also possible that the introduced crop trees were less well-adapted to the prevalent soil conditions than the native tree species and accumulated phytotoxic concentrations of Al and Fe over the years. As a consequence, root health may decline and root soil exploration and root litter input into soil decrease, thereby, eventually leading to alterations in soil properties. To disentangle the underlying mechanisms, experimental studies with mixtures of oil palm, rubber and native forest species are necessary. Thereby, feed-back effects between ecosystem functions and functional traits of distinct tree species and their communities can be uncovered and used to develop improved management strategies.
